# Liver biopsy as a useful diagnostic tool for hepatic sarcoidosis: A case report

**DOI:** 10.3892/mi.2024.162

**Published:** 2024-05-14

**Authors:** Kenrei Uehara, Tatsuo Kanda, Shuhei Arima, Mai Totsuka, Masayuki Honda, Ryota Masuzaki, Reina Sasaki-Tanaka, Naoki Matsumoto, Masahiro Ogawa, Hirofumi Kogure

**Affiliations:** Division of Gastroenterology and Hepatology, Department of Medicine, Nihon University School of Medicine, Tokyo 137-8610, Japan

**Keywords:** hepatic sarcoidosis, liver biopsy, angiotensin-converting enzyme, gallium scintigraphy

## Abstract

In certain cases, it is difficult to distinguish hepatic sarcoidosis from malignant lymphoma or drug-induced liver injury and to select the proper treatment for this condition. The present study describes the case of a female patient in her 30s who was referred to the hospital due to fever, arthralgia, myalgia and abnormal liver function test results for 4 months. A laboratory examination revealed elevated levels of serum angiotensin-converting enzyme (ACE) and soluble interleukin-2 receptor (sIL-2R), as well as an increase in serum hepatic and biliary tract enzymes. Gallium scintigraphy revealed a marked uptake in the liver, as well as an uptake in the mediastinal, inguinal and external iliac lymph nodes. Magnetic resonance imaging revealed extensive hepatosplenomegaly with multiple non-enhancing splenic nodules. Hepatic sarcoidosis was diagnosed by a liver biopsy as non-caseating hepatic granulomas, and multinucleated giant cells were observed. The patient responded to treatment with 20 mg prednisolone daily, and exhibited an improvement in her symptoms. An improvement was also observed in her serum levels of ACE, sIL-2R, and serum hepatic and biliary tract enzymes; decreased gallium uptake in the liver was also observed. On the whole, the present case report reconfirms that liver biopsy is a useful diagnostic tool for hepatic sarcoidosis.

## Introduction

Sarcoidosis, a disease of unknown etiology, is a systemic granulomatous disorder. Pulmonary hypertension, respiratory failure from pulmonary fibrosis, as well as complications due to the involvement of other organs, including cardiac, neurologic and hepatic involvement, and hemoptysis from aspergilloma, are major causes of mortality in patients with sarcoidosis (1). Sarcoidosis is one of the most common causes of non-caseating hepatic granulomas (2).

There are several biomarkers used for sarcoidosis (3,4). Angiotensin-converting enzyme (ACE) is expressed in several human tissues, including the lungs, as a membrane-bound acid glycoprotein mostly produced by activated alveolar macrophages, and ACE converts angiotensin I to angiotensin II (3,5). A meta-analysis demonstrated that the serum ACE concentration could assist in the diagnosis of sarcoidosis and the prediction of sarcoidosis activity, despite the cautious interpretation of the serum ACE concentration results (3).

The soluble interleukin (IL)-2 receptor (sIL-2R) is also used as a biomarker for determining disease severity in patients with sarcoidosis (4). IL-2 is involved in immunity and the functions of sIL-2R are controversial and vary from activation to the inhibition of immunity (6). Measuring sIL-2R levels is useful for the diagnosis of hemophagocytic lymphohistiocytosis, the follow-up of patients with granulomatous diseases and autoimmune diseases, and for the prognosis of malignant diseases (6). It has been reported that sIL-2R has a high performance as an adjunctive diagnostic marker for lymphoma, particularly among patients with fever (7).

The present study describes the case of a patient with hepatic sarcoidosis for whom the liver biopsy proved useful for diagnosis. The case presented herein reconfirms that liver biopsy is a useful diagnostic tool for hepatic sarcoidosis.

## Case report

A woman in her 30s was referred to the local hospital near her residence due to a fever of 38-40˚C, arthralgia, myalgia and abnormal liver function test results for 4 months prior to her first visit to Nihon University Itabashi Hospital (Tokyo, Japan).

She was regularly observed by a local doctor for obsessive-compulsive disorder and panic disorder, and she was on several medications. She had a history of allergy to escitalopram. She did not consume alcohol and had no history of surgery, transfusion, tattooing or drug abuse. She had no family history of liver disease.

Upon her first visit, the patient*'*s body length and weight were 160 cm and 60 kg, respectively. Her blood pressure, pulse rate, O2 saturation and body temperature were 110/73 mmHg, 75/min, 98% and 37.0˚C, respectively. She was conscious, her conjunctiva were not icteric or pale, and her liver was elastic, hard and palpable when using three horizontal fingers to palpate the epigastric and right hypochondrium regions. No edema was observed in her feet.

A laboratory examination revealed elevated serum ACE and sIL-2R levels, as well as an increased in serum hepatic and biliary tract enzymes (Table I). The serum calcium concentration was within the normal limits. The C-reactive protein (CRP) level was elevated, indicating inflammation. As the levels of the protein induced by vitamin K absence or antagonist II (PIVKA-II) was elevated, screening for hepatocellular carcinoma (HCC) was also performed.

**Figure 1:**
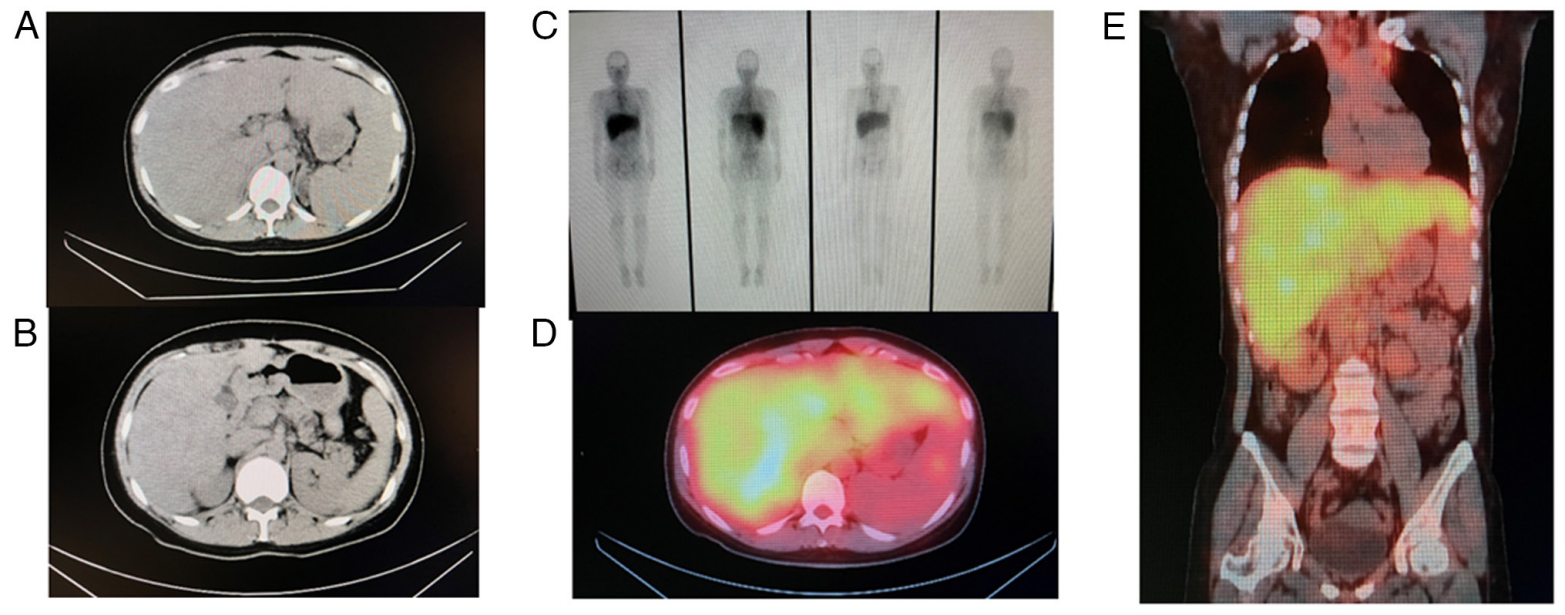
Pre-treatment CT scan and gallium-67 scintigraphy. (A and B) An abdominal CT scan revealed extensive hepatosplenomegaly. (C-E) Gallium-67 scintigraphy demonstrated a marked uptake in the liver and uptake in the mediastinal, inguinal and external iliac lymph nodes. CT, computed tomography.

**Figure 2:**
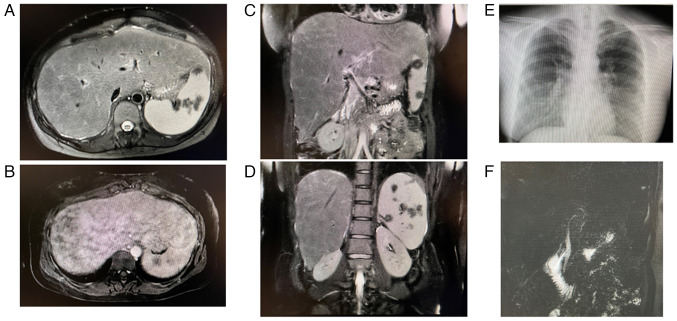
Pre-treatment MRI and posteroanterior chest X-ray. (A-D) MRI revealed extensive hepatosplenomegaly with multiple non-enhancing splenic nodules. (A, C and D) T2-weighted fat saturation-MRI; (B) gadolinium-ethoxybenzyl-diethylene-triaminepentaacetic acid-MRI late-stage. (E) A chest X-ray displayed no abnormal shadows in the lung field. (F) Magnetic resonance cholangiopancreatography demonstrated no stenosis or obstruction of the intrahepatic or extrahepatic bile ducts. MRI, magnetic resonance imaging.

A computed tomography (CT) scan revealed the swelling of the mediastinal lymph nodes and extensive hepatosplenomegaly (Fig. 1A and B). Gallium scintigraphy demonstrated a marked uptake in the liver, as well as an uptake in the mediastinal, inguinal and external iliac lymph nodes (Fig. 1C-E). Magnetic resonance imaging (MRI) indicated extensive hepatosplenomegaly with multiple non-enhancing splenic nodules (Fig. 2A-D). A posteroanterior chest X-ray displayed no abnormal shadows in the lung field (Fig. 2E). Magnetic resonance cholangiopancreatography demonstrated no stenosis or obstruction of the intrahepatic or extrahepatic bile ducts (Fig. 2F). No HCCs were detected by imaging modalities.

**Figure 3:**
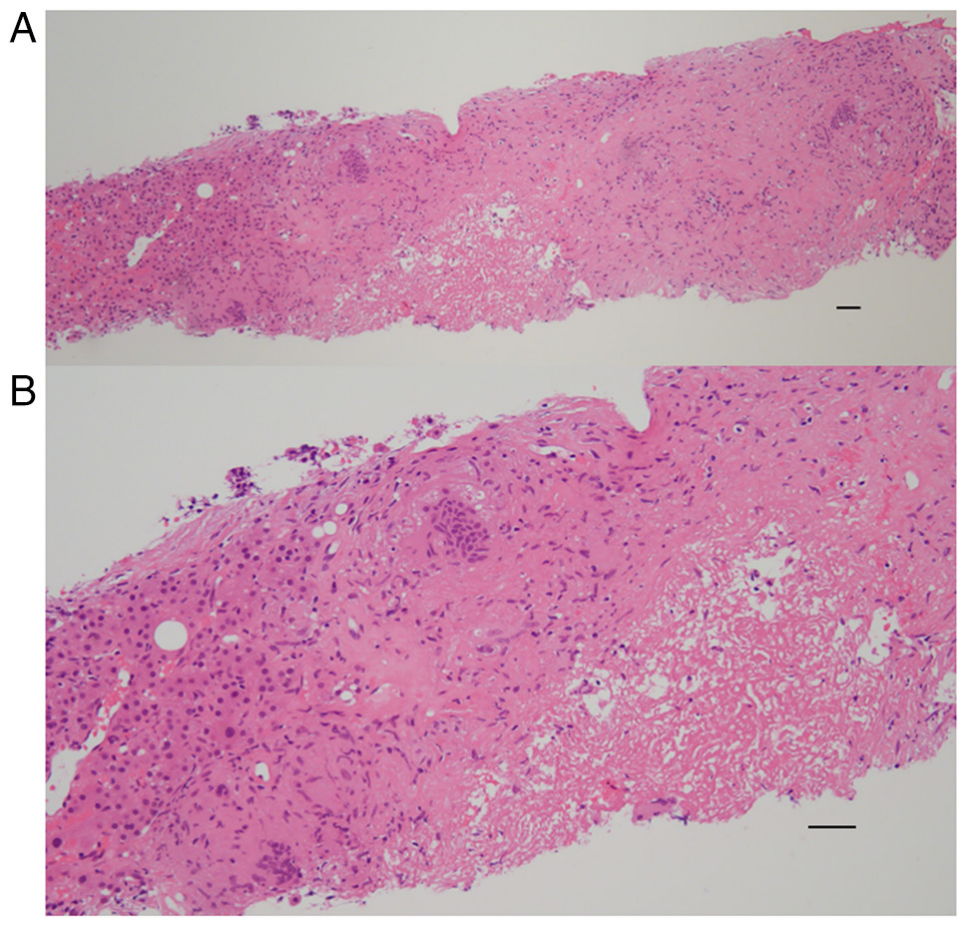
Pre-treatment pathological findings of the liver biopsy. (A) The hepatic architecture was slightly disrupted, but no cirrhosis was observed (hematoxylin and eosin staining; magnification, x10). Scale bar, 200 µm. (B) Non-caseating hepatic granulomas and multinucleated giant cells were observed (hematoxylin and eosin staining; magnification, x40). Scale bar, 100 µm.

**Figure 4:**
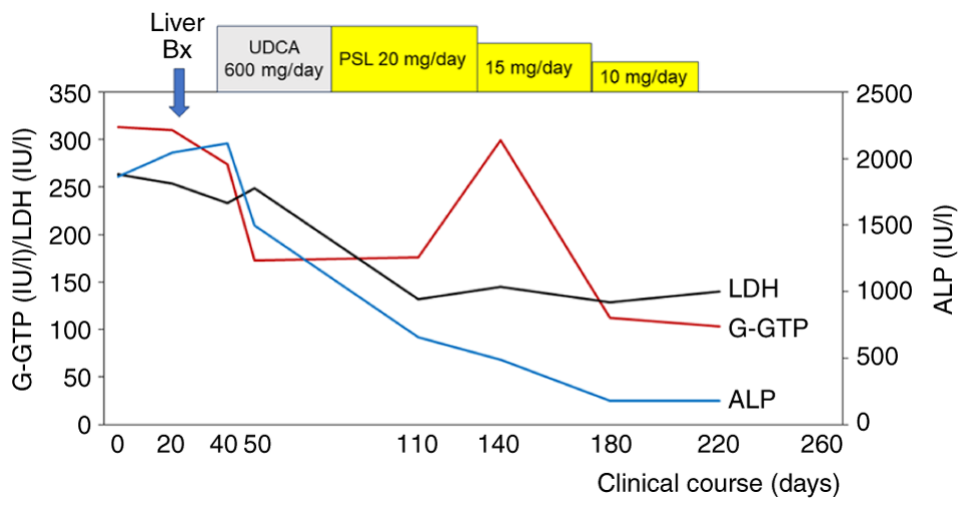
The clinical course and changes in the levels of hepatobiliary enzymes in the patient described herein. Liver Bx, liver biopsy; UDCA, ursodeoxycholic acid; PSL, prednisolone; G-GTP, γ-glutamyl transpeptidase; LDH, lactate dehydrogenase; ALP, alkaline phosphatase.

A liver biopsy was performed to differentially diagnose the cause of liver disease and the liver specimen, including hematoxylin and eosin staining, was prepared and performed at the Department of Pathology, Nihon University Itabashi Hospital (Tokyo, Japan) (Fig. 3). Hepatic sarcoidosis was diagnosed due to the observation of non-caseating hepatic granulomas and multinucleated giant cells (2). Following the diagnosis of hepatic sarcoidosis, the patient was first treated with 600 mg daily ursodeoxycholic acid (UDCA) (8,9); however, due to the development of diarrhea, treatment with UDCA was terminated. Thus, treatment with 20 g prednisolone daily was commenced, and an improvement in her symptoms and in the serum levels of ACE (from 55.7 to 7.2 U/l), sIL-2R (from 2,380 to 330 U/ml) and serum hepatic and biliary tract enzymes was observed (Fig. 4).

**Figure 5:**
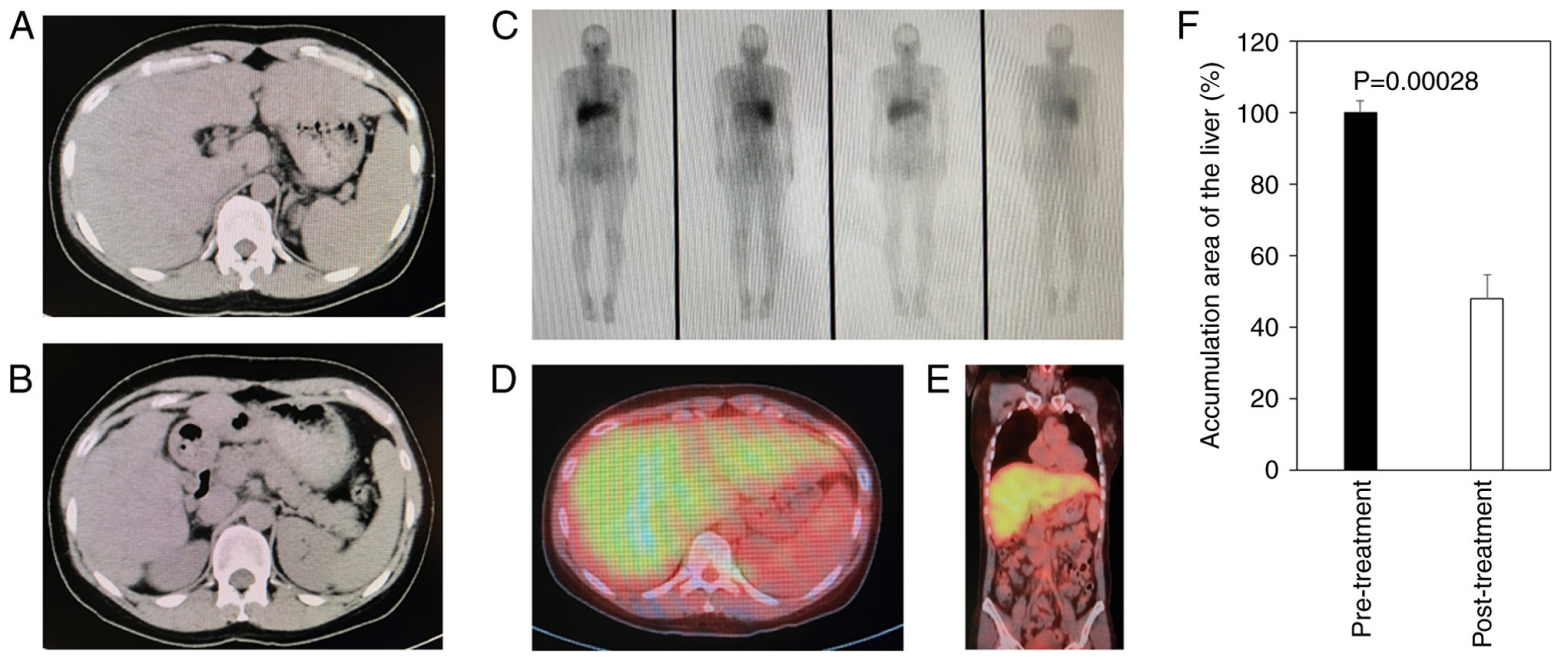
Three-month post-treatment CT scan and gallium-67 scintigraphy. (A and B) An abdominal CT scan revealed improved hepatosplenomegaly; (C-E) the decreased uptake of gallium-67 in the liver was observed. (F) Gallium scintigraphy demonstrated the accumulation area in the liver at post-treatment (n=3; Fig. 5C, liver in only left panel) significantly decreased, compared to that at pre-treatment (n=3, Fig. 1C, liver in only left panel). Each image was analyzed three times, and the Student*'*s t-test was used for statistical analysis. The results were considered statistically significant at P<0.05.

Following treatment, a CT scan revealed an improvement in hepatosplenomegaly, and a decrease in gallium uptake in the liver was observed (Fig. 5A and B). Compared with those at the pre-treatment CT scan (Fig. 1A and B), the hepatomegaly and gallium uptake in the liver decreased at 3 months post-treatment. The accumulation area of gallium-67 decreased in the liver (Fig. 5C-F). Each image was analyzed three times, and the Student*'*s t-test was used for statistical analysis.

At 1 year after her first visit to Nihon University Itabashi Hospital (Tokyo, Japan), although she still used 7.5 mg daily prednisolone, 50 mg azathioprine daily and 600 mg UDCA daily, the patient felt well with lower serum levels of hepatic and biliary tract enzymes (aspartate aminotransferase, 40 IU/l; alanine aminotransferase, 36 IU/l; lactate dehydrogenase, 163 IU/l; alkaline phosphatase, 182 IU/l; and γ-glutamyl transpeptidase, 44 IU/l) and PIVKA-II (26 mAU/ml).

## Discussion

In certain cases, it may be difficult to differentially diagnose hepatic sarcoidosis, drug-induced liver injury, malignant lymphoma, viral hepatitis or other liver diseases. Moreover, it is difficult to select a treatment strategy for patients with hepatic sarcoidosis. In the case present herein, after the liver biopsy was performed, the diagnosis of hepatic sarcoidosis was confirmed and the patient was then treated with UDCA and/or prednisolone.

It has been shown that a liver biopsy reveals hepatic involvement in 12-90% of patients with sarcoidosis (10-12). Although a number of patients with hepatic sarcoidosis are asymptomatic, some patients exhibit various symptoms (13,14), such as portal hypertension and intrahepatic cholestasis. In the case in the present study, an endoscopy did not reveal any varices of the stomach or esophagus (data not shown).

Israel *et al* (15) reported that serum ACE levels and gallium scans were helpful for the diagnosis of hepatic sarcoidosis, although the etiology of hepatic granulomatosis discovered among patients with normal chest X-rays is controversial. Inoue *et al* (16) reported that a marked uptake in gallium scintigraphy was observed in patients with hepatic sarcoidosis, suggesting that the patient described herein had hepatic sarcoidosis.

Graf *et al* (9) reported that 62 patients with hepatic involvement (4.2%) among 1,476 patients with sarcoidosis were identified, and that 54 patients with hepatic sarcoidosis were medically treated, most commonly with glucocorticoids (69.4%) or UDCA (40.3%). ALP levels decreased by 60.8% or 59.9% on average from baseline in patients treated with glucocorticoids or those treated with UDCA, respectively (9). Kikuchi *et al* (17) reported that the treatment of a patient with hepatic sarcoidosis using both corticosteroids and UDCA resulted in marked improvements biochemically and histologically. In the patient described herein, azathioprine was also used to reduce the dose of prednisolone (18-20). However, further studies may be required to confirm the use of these for hepatic sarcoidosis.

In the case presented herein, as various symptoms appeared and abnormal liver function test results were observed, a liver biopsy was performed. After hepatic sarcoidosis was diagnosed, the administration of prednisolone could have led to improvements in the subjective symptoms and abnormal liver function tests of the patient. The authors aim to carefully follow-up the patient in the future.

Abdominal imaging, such as ultrasound and MRI, of the hepatic and splenic sarcoidosis, indicates contour irregularity, nodularity and abnormal signal intensity (21-23). Patients with hepatic sarcoidosis have hyperechoic, heterogeneous, or nodular livers in the ultrasound (21). These findings are not specific for sarcoidosis and chronic hepatitis or cirrhosis. An MRI also demonstrates severe fibrosis of the liver parenchyma with contour irregularity and linearly enhanced foci (23). It may be difficult to diagnose hepatic sarcoidosis only by abdominal imaging modalities.

In the case in the present study, the levels of immunoglobulin G4, lysozyme or M protein were not measured. The levels of serum immunoglobulin G4, lysozyme or M protein occasionally increase in hepatic sarcoidosis and their measurement may be useful for ruling out other diseases (16,24,25).

In the case described herein, tuberculosis was also ruled out by the liver biopsy and the negative result of interferon-gamma releasing assay for the diagnosis of active tuberculosis (data not shown) (26). Although the use of a liver biopsy may not a novel methodology, it appears useful as a diagnostic tool for hepatic sarcoidosis.

In cases with unknown abnormal liver function tests, liver sarcoidosis should be suspected and thus serum ACE levels should be measured. In conclusion, as demonstrated herein, liver biopsy is required to diagnose hepatic sarcoidosis in patients with more severe, yet subjective symptoms and inflammation. However, further case and long-term follow-up data of similar cases are required to fully confirm the findings. Additional studies with larger patient cohorts are also required to validate the findings.

## Figures and Tables

**Table I:** Laboratory data of the patient upon her first visit to the hospital.

Item (units)	Values	Normal range	Item (units)	Values	Normal range
**WBC (x10^3^/µl)**	**5.9**	3.3-8.6	**TP (g/dl)**	**8.1**	6.6-8.1
**Hemoglobin (g/dl)**	**14.1**	11.6-14.8	Albumin (g/dl)	3.6	4.1-5.1
**Platelets (x10^3^/µl)**	**162**	158-348	**BUN (mg/dl)**	**7.1**	8-20
**Neutrophils (%)**	**66.3**	40-70	**Creatinine (mg/dl)**	**0.60**	0.46-0.79
**Basophils (%)**	**0.5**	0-2	**Calcium (mg/dl)**	**9.2**	8.8-10.1
**Eosinophils (%)**	**3.4**	0-5	CRP (mg/dl)	3.81	<0.2
**Monocytes (%)**	**6.7**	2-10	ESR (mm/1 hr)	52	3-15
Lymphocytes (%)	23.1	25-55	sIL-2R (U/ml)	2,380	157-474
**PT (%)/INR**	**100/0.99**	80</0.9-1.1	ACE (U/l)	55.7	8.3-21.4
**T. CHO (mg/dl)**	**200**	142-219	**ANA**	**<40**	<40
**TG (mg/dl)**	**112**	30-149	IgG (mg/dl)	2,083	861-1747
**Glucose (mg/dl)**	**86**	73-109	IgA (mg/dl)	513	93-393
**HbA1c (%)**	**6.1**	4.6-6.2	**IgM (mg/dl)**	**137**	50-269
AST (IU/l)	56	13-30	M2BPGi (COI)	2+/3.98	-/1.00
ALT (IU/l)	36	7-23	**AFP (ng/ml)**	**2.4**	<20
LDH (IU/l)	263	124-222	PIVKA-II (mAU/ml)	84	<40
ALP (IU/l)	1863	106-322	**NH3 (µg/dl)**	**50**	20-80
γ-GTP (IU/l)	313	9-32	**Anti-HIV**	**Negative**	Negative
**CPK (U/ml)**	**31**	41-153	**HBsAg**	**Negative**	Negative
**T. Bil (mg/dl)**	**0.69**	0.4-1.5	**anti-HBc**	**Negative**	Negative
**D. Bil (mg/dl)**	**0.37**	0.1-0.4	**anti-HCV**	**Negative**	Negative

Items with values within the normal limits are indicated in bold font. WBC, white blood cell count; PT, prothrombin time; INR, international normalized ratio; T. CHO, total cholesterol; TG, triglyceride; HbA1c, hemoglobin A1c; AST, aspartate aminotransferase; ALT, alanine aminotransferase; LDH, lactate dehydrogenase; ALP, alkaline phosphatase; γ-GTP, γ-glutamyl transpeptidase; CPK, creatine phosphokinase; T. Bil, total bilirubin; D. Bil, direct bilirubin; TP, total protein; BUN, blood urea nitrogen; CRP, C-reactive protein; ESR, erythrocyte sedimentation rate; sIL-2R, soluble interleukin-2 receptor; ACE, angiotensin-converting enzyme; ANA, anti-nuclear antibody; Ig, immunoglobulin; M2BPGi, macrophage galactose-specific lectin-2 binding protein glycosylation isomer; AFP, alpha-fetoprotein; PIVKA-II, protein induced by vitamin K absence or antagonist II; NH3, ammonia; anti-HIV, anti-human immunodeficiency virus antibody; HBsAg, hepatitis B surface antigen; anti-HBc, anti-hepatitis B core antibody; anti-HCV, anti-hepatitis C virus antibody; COI, cut off index.

## Data Availability

The data sets used and/or analyzed during the current study are available from the corresponding author upon reasonable request.
